# Patient-specific image-based bone marrow dosimetry in Lu-177-[DOTA^0^,Tyr^3^]-Octreotate and Lu-177-DKFZ-PSMA-617 therapy: investigation of a new hybrid image approach

**DOI:** 10.1186/s13550-018-0427-z

**Published:** 2018-08-03

**Authors:** Astrid Gosewisch, Andreas Delker, Sebastian Tattenberg, Harun Ilhan, Andrei Todica, Julia Brosch, Lena Vomacka, Anika Brunegraf, Franz Josef Gildehaus, Sibylle Ziegler, Peter Bartenstein, Guido Böning

**Affiliations:** Department of Nuclear Medicine, University Hospital, LMU Munich, Marchioninistrasse 15, 81377 Munich, Germany

**Keywords:** Radionuclide therapy, Bone marrow, Dosimetry, Hybrid imaging, Lutetium-177, Prostate cancer, PSMA, mCRPC, Neuroendocrine tumour, Octreotate, NET

## Abstract

**Background:**

The bone marrow (BM) is a main organ at risk in Lu-177-PSMA-617 therapy of prostate cancer and Lu-177-Octreotate therapy of neuroendocrine tumours. BM dosimetry is challenging and time-consuming, as different sequential quantitative measurements must be combined. The BM absorbed dose from the remainder of the body (ROB) can be determined from sequential whole-body planar (WB-P) imaging, while quantitative Lu-177-SPECT allows for more robust tumour and organ absorbed doses. The aim was to investigate a time-efficient and patient-friendly hybrid protocol (HP) for the ROB absorbed dose to the BM. It combines three abdominal quantitative SPECT (QSPECT) scans with a single WB-P acquisition and was compared with a reference protocol (RP) using sequential WB-P in combination with sequential QSPECT images. We investigated five patients receiving 7.4 GBq Lu-177-Octreotate and five patients treated with 3.7 GBq Lu-177-PSMA-617. Each patient had WB-P and abdominal SPECT acquisitions 24 (+ CT), 48, and 72 h post-injection. Blood samples were drawn 30 min, 80 min, 24 h, 48 h, and 72 h post-injection. BM absorbed doses from the ROB were estimated from sequential WB-P images (RP), via a mono-exponential fit and mass-scaled organ-level *S* values. For the HP, a mono-exponential fit on the QSPECT data was scaled with the activity of one WB-P image acquired either 24, 48, or 72 h post-injection (HP24, HP48, HP72). Total BM absorbed doses were determined as a sum of ROB, blood, major organ, and tumour contributions.

**Results:**

Compared with the RP and for Lu-177-Octreotate therapy, median differences of the total BM absorbed doses were 13% (9–17%), 8% (4–15%), and 1% (0–5%) for the HP24, HP48, and HP72, respectively. For Lu-177-PSMA-617 therapy, total BM absorbed doses deviated 10% (2–20%), 3% (0–6%), and 2% (0–6%).

**Conclusion:**

For both Lu-177-Octreotate and Lu-177-PSMA-617 therapy, BM dosimetry via sequential QSPECT imaging and a single WB-P acquisition is feasible, if this WB-P image is acquired at a late time point (48 or 72 h post-injection). The reliability of the HP can be well accepted considering the uncertainties of quantitative Lu-177 imaging and BM dosimetry using standardised organ-level *S* values.

## Background

Over the recent years, radionuclide therapy using Lu-177-Octreotate and Lu-177-PSMA-617 evolved as a promising approach for the treatment of metastasised and inoperable neuroendocrine tumours (NET) and metastasised, castration-resistant prostate cancer (mCRPC), respectively [1–3]. The red or active bone marrow (BM) represents a main organ at risk in radionuclide therapy [4–8]*.* Bone marrow toxicity is particularly of concern in Lu-177-PSMA-617 therapy, as patients suffering from mCRPC often present with a high burden of bone metastases. The latter may cause pronounced activity accumulations in close proximity to the regions which potentially bear active marrow. At these locations, especially the *γ*-component of the Lu-177 decay might lead to a significant photon cross-irradiation of the bone marrow [9]. However, for Lu-177-Octreotate therapy, the bone marrow is also considered as an organ at risk, as patients with progressive cancer disease usually already underwent several pre-therapies such as external radiotherapy or chemotherapy [3, 4]. These pre-therapies may have interfered with the haematological function of the bone marrow. Thus, bone marrow dosimetry is highly recommended in these patients for risk reduction of marrow toxicities and, at the same time, an as high as possible tumour absorbed dose [10].

The total bone marrow absorbed dose is composed of different contributions originating from various activity source regions: (1) the bone marrow self-absorbed dose including the active bone marrow cells, the extracellular fluid, and the blood cells; (2) activity accumulations in the remaining skeleton composed of compact bone or fatty tissue (yellow or inactive marrow); (3) the cross-absorbed dose by major organs or tumours; and (4) the cross-irradiation coming from the remainder of the body (ROB; whole body minus specific or unspecific accumulations in the other source regions) [11]. Each absorbed dose component requires a dedicated measurement procedure to derive its respective time-activity curve (TAC) and the source-specific time-integrated activity. The cumulated actvity-to-absorbed-dose conversion is usually performed via pre-calculated and standardised organ-level *S* values [11].

The appropriate data collection to accurately quantify the various possible source regions is challenging and leads to both a high clinical workload and long patient examination times, if bone marrow dosimetry shall be routinely performed in the clinic. For Lu-177-Octreotate or Lu-177-PSMA-617 therapy, the bone marrow absorbed dose from the major accumulating organs (*D*_BM ← organs_), the ROB (*D*_BM ← ROB_), and the blood (*D*_BM ← blood_) can be determined from sequential quantitative SPECT images, sequential quantitative whole-body planar images, and multiple blood samples, respectively, in combination with the corresponding *S* values [8, 9, 11–13]. However, despite the high metastatic load which might be observed for NET and mCRPC patients, it is challenging to explicitly consider the bone marrow absorbed dose from activity accumulations in the tumours (*D*_BM ← tumours_) via standardised and pre-calculated tumour-to-bone marrow *S* values, as the latter intrinsically cannot consider the large inter-patient variability of the shape, size, and distribution of all lesions [14].

Our institutional protocol determines the absorbed dose contribution from the ROB via sequential whole-body planar images [11], which are acquired at three time points at 24, 48, and 72 h post-injection. In addition, we decided to derive organ (e.g. kidneys) and tumour absorbed doses from sequential quantitative SPECT measurements for improved organ and tumour dosimetry [15–18]. However, full whole-body quantitative Lu-177 SPECT is still not commonly used in the clinic, implicating the need of consecutive planar and SPECT imaging at each time point to obtain both reliable bone marrow absorbed doses from the ROB and reliable organ or tumour absorbed doses [19]. Particularly, the increased examination time in case of consecutive SPECT and whole-body planar imaging leads to an increased clinical workload and patient discomfort, as patients with progressive cancer disease may suffer from a bad health condition. Thus, the aim of this work was to derive a time-efficient, patient-friendly, and simplified bone marrow dosimetry protocol for clinical routine. Therefore, we investigated the possibility to reduce the number of image acquisitions from three whole-body planar and three quantitative SPECT scans (reference protocol (RP)) to a single whole-body planar acquisition while maintaining the institution’s usual sequential quantitative SPECT protocol (hybrid protocol (HP)). Further, we investigated the effect of this image reduction on the bone marrow absorbed dose from the ROB and on the total bone marrow dose (*D*_BM ← total_), to prove whether the proposed hybrid protocol provides comparable absorbed dose estimates for both Lu-177-Octreotate and Lu-177-PSMA-617 therapy. For the determination of the total bone marrow absorbed dose, the energy depositions in the bone marrow due to activity accumulations in the ROB, blood, major organs, and tumours were considered. Furthermore, we determined the best-suited time point for this single whole-body planar image acquisition with respect to the time points available in our institutional protocol. All absorbed dose calculations are based on the organ-level *S* values (e.g. whole ROB to bone marrow) [11].

## Methods

### Patient selection, data acquisition, and image quantification

#### Patient selection

This study is based on ten patients, with five patients suffering from somatostatin receptor-positive neuroendocrine metastases (P1-P5) and five patients from mCRPC with expression of PSMA-avid lesions (P6-P10). Details for each patient are provided in Tables 1 and 2. All patients received multiple therapy cycles of approximately 3.7 GBq Lu-177-DKFZ-PSMA-617 (Lu-177-PSMA-617) or 7.4 GBq Lu-177-[DOTA^0^,Tyr^3^]-Octreotate (Lu-177-Octreotate). All patients except one mCRPC patient showed soft tissue lesions on the pre-therapeutic Ga-68-HBED-CC-PSMA or Ga-68-[DOTA^0^,Tyr^3^]-Octreotate PET/CT scans, while all prostate-specific membrane antigen (PSMA) patients and two NET patients additionally presented with bone metastases (Tables 1 and 2). The local ethics committee approved the study protocol and did not desire any written consent for the study entry. The study is based on retrospective and anonymised patient data.

**Table 1:** Characteristics of all NET patients included in this study for Lu-177-Octreotate therapy

Octreotate	P1	P2	P3	P4	P5
Sex	M	M	F	F	F
Age	68	66	61	47	73
Activity investigated cycle [MBq]	7654	7425	7420	7409	7410
Diagnosis	NET small intestine	NET	NET terminal ileum	NET pancreas	NET stomach
Metastases (PET/CT)					
- Extend	Medium	Medium	Medium	Medium	Medium
- Type (VIS = visceral, LYM = lymph, OSS = osseous)	Mainly VIS (liver), LYM	Mainly LYM, VIS (liver)	Mainly VIS (liver), LYM, OSS	Mainly LYM, VIS (liver and other)	Mainly VIS (liver), OSS
Proliferation index	Ki67 3–4%	Ki67 5–10%	Ki67 1%	Ki67 10%	Ki67 10%
Pre-therapies	SSA-analogues	Interferon alpha	Hemicolectomy, SSA-analogues, radioembolization	Chemotherapy (stroptozotocin/5-FU, dacarbazapin, capecitabin/Te-modal)	SSA-analogues, bisphosphonate therapy
Blood pre-therapy					
- Leukocytes [G/l]	7.76	10.4	3.79	3.93	10.2
- Erythrocytes [T/l]	4.58	4.50	4.43	3.73	4.42
- Thrombocytes [G/l]	207	294	297	177	303
- Haematocrit	0.421	0.442	0.373	0.341	0.399

**Table 2:** Characteristics of all mCRPC patients included in this study for Lu-177-PSMA-617 therapy

PSMA-617	P6	P7	P8	P9	P10
Age	68	66	61	47	73
Activity investigated cycle [MBq]	3718	3743	3745	3752	3700
Diagnosis	mCRPC	mCRPC	mCRPC	mCRPC	mCRPC
Metastases (PET/CT)					
- Extend	High	High	High	High	High
- Type (VIS = visceral, LYM = lymph, OSS = osseous)	Mainly VIS (liver), OSS, LYM	Only OSS	Mainly OSS, LYM	Mainly OSS, LYM	Mainly OSS, LYM
Initial TNM classification	pT3b, pN1, R0, G3, Gleason 9	pT4, N1, R1, G3, Gleason 8	pT3b, pN1, R1, Gleason 9	pT3a, pN1, pR1, Gleason 9	pT4, N1, R1, Gleason 9
PSA [ng/ml]	368	1201	5436	408	101
Pre-therapies (1, yes/0, no)					
- Surgery	0	1	1	1	1
- Radiotherapy	1	1	1	0	0
- Anti-hormonal therapy (including bicalutamide, enzalutamide, abiraterone acetate)	1	1	1	1	1
- Radionuclide therapy (Ra-223)	0	1	0	1	1
- Chemotherapy (docetaxel, cabazitaxel)	1	1	1	1	0
Blood pre-therapy					
- Leukocytes [G/l]	4.90	7.20	5.60	6.20	5.00
- Erythrocytes [T/l]	4.31	4.79	4.08	4.35	4.00
- Thrombocytes [G/l]	307	195	291	314	191
- Haematocrit	0.376	0.406	0.335	0.377	0.366

#### Data acquisition

Data for dosimetry were acquired during a routine 4-day in-patient stay following the radiopharmaceutical injection, in conjunction with standard clinical examinations. All patients received a 15-min one-bed abdominal SPECT scan and a 20-min whole-body planar scintigraphy at 24, 48, and 72 h post-injection (p. i.) on a dual-headed Symbia T2 SPECT/CT (Siemens Medical Solutions, Erlangen, Germany). Counts were detected for the photopeak window of 208 keV (width 15%) by the usage of a medium-energy low-penetration collimator. Two additional scatter windows were measured at 170 keV (width 15%) and 240 keV (width 10%). A low-dose AC-CT was acquired at the first image acquisition session for anatomical correlation and attenuation correction during quantitative SPECT reconstruction. For the determination of the absorbed dose to the bone marrow from the activity circulating in the blood, five venous blood samples were drawn from the site contralateral to injection at 30 and 80 min p. i. and 24, 48, and 72 h p. i. [9, 20].

#### SPECT image reconstruction and quantification

Quantitative SPECT images were reconstructed as described by Delker et al. [9] via a rotation-based, penalised, one-step-late ordered subset expectation maximisation algorithm, which included corrections for scatter, attenuation, and distance-dependent geometrical collimator blur. Attenuation correction was performed for each SPECT scan via the AC-CT, which was acquired along with the SPECT scan 24 h post-injection. To apply the attenuation correction, especially to the SPECT scans 48 and 72 h p. i., the single AC-CT was co-registered onto an initial SPECT reconstruction without attenuation correction by using a rigid body co-registration algorithm with six degrees of freedom (PMOD Version 3.609, PMOD Technologies, Zurich, Switzerland). If only one AC-CT is acquired for sequential SPECT imaging, special care has to be taken to minimise misregistration between SPECT images and separately acquired CT scans, as such a misalignment can distort the proper attenuation correction and, thus, activity quantification. This is in principal also true for serial SPECT and CT imaging, as even within a single image acquisition session patient movements cannot be entirely avoided. Scatter correction employed the triple energy window (TEW) method. Correction for distance-dependent collimator blur made use of a Gaussian blur model. Corrections for partial volume effects and dead time were not applied. For conversion of the measured counts per second and per voxel to Becquerel per millilitre, an appropriate calibration factor was determined. Therefore, we used a large cylinder of approximately 20 cm diameter, which was filled with a known activity concentration and which has been imaged and reconstructed via the same protocol [9, 15, 20].

#### Planar image correction and calibration

**Fig. 1:**
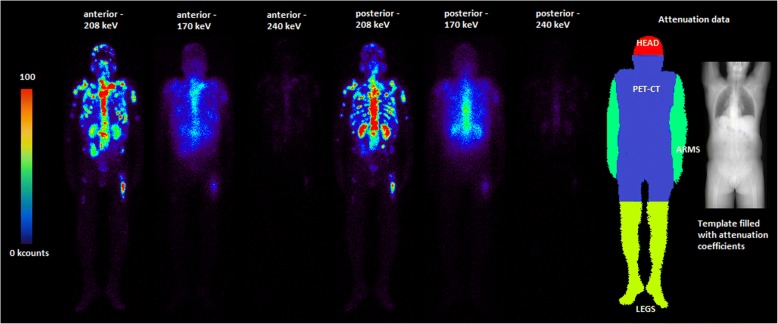
Workflow for planar image quantification shown for patient P8. Anterior and posterior views for all three energy windows (24 h p. i.; photopeak and both scatter windows) and attenuation map based on the projected patient-specific CT from the PET/CT scan

For each patient, all acquired whole-body planar images were corrected for scatter and attenuation on a pixel basis via a dedicated MATLAB routine (Fig. 1) [16, 20, 21]; for the correction of scatter, the TEW method was applied, as for the quantitative SPECT images. For the attenuation correction, a linear projection of *μ* values along the ventral axis of the patient was created from the diagnostic CT image of the pre-therapeutic Ga-68 PET/CT scan, which covered nearly the whole patient body from the middle of the head to approximately the knees. Therefore, a conversion between the Hounsfield units (HUs) in the diagnostic CT and the *μ* values at 208 keV was established by acquiring a CT scan of a Gammex tissue phantom (Gammex 467; Gammex, Inc., Middleton, WI) with 16 tissue rods of known composition and thus known attenuation characteristics [22]. The *μ* values of all rods were plotted against the measured HUs, and a bilinear fit model (range 1: HU = (− 688;0); range 2: HU = (0;1127)) was applied to the whole data set [15, 22]. This calibration curve allows for the assignment of *μ* values to a continuous range of HUs. The lower arms and legs as well as a part of the head were not included in the PET/CT data, as the arms are usually positioned above the head during the PET/CT scan and as the PET/CT scan is usually not acquired over the entire patient length. By contrast, the arms, legs, and head are fully included in the whole-body planar images, and an appropriate *μ* value has to be defined for each segment (Fig. 1). Thus, mean *μ* values derived from three patients with PET/CT acquisitions of the head, legs, and arms were assigned to the missing segments. Therefore, all segments—the part visible on the PET/CT and the missing parts of the head, arms, and legs—were delineated on the co-registered whole-body planar images (delineation and rigid body co-registration via PMOD Version 3.609). The resulting map of the regions of interest (ROI) was saved, with each ROI segment being characterised via a defined value. This ROI map was then loaded by a self-designed MATLAB routine, which assigned the defined *μ* value to each segment according to the ROI number. Afterwards, the resulting whole-body integral μ-map was blurred via a Gaussian filter with a width approximating an average resolution of the gamma camera (geometric resolution of full width at half maximum of 11 mm at 10 cm). Pixel-wise attenuation correction was finally performed in conjunction with geometric averaging of both planar views (conjugate view method) [21].

Calibration of whole-body planar images was performed via the corresponding quantitative abdominal SPECT by using the fact that ideally the total activity *A*_SPECT_ within the quantitative SPECT should be correlated to the number of counts per second (cps) *x*_planar_ in the planar abdominal counterpart multiplied by an appropriate calibration factor:

1 $$ {A}_{\mathrm{SPECT}}={x}_{\mathrm{planar}}\bullet {C}_{\mathrm{planar},\mathrm{SPECT}}. $$

*C*_planar, SPECT_ denotes the SPECT-based calibration factor in units of Bq/cps.

### Bone marrow dosimetry formalism

To derive total bone marrow absorbed doses (*D*_BM ← total_), a sum of the bone marrow self-absorbed dose from the activity in the blood (*D*_BM ← blood_) as well as the bone marrow cross-absorbed dose by major organs (*D*_BM ← organs_), tumours (*D*_BM ← tumours_), and the ROB (*D*_BM ← ROB_) was considered according to the findings of previous studies [5, 7–9, 13]. If not indicated otherwise, the general term bone marrow always refers to the red or active bone marrow, i.e. the radiation-sensitive part of the bone marrow mixture [23]. The dose contribution of each source component to the bone marrow was estimated according to the guidelines of the European Association of Nuclear Medicine (EANM) [11]. Following the Medical Internal Radiation Dose (MIRD) formalism underlying the EANM guidelines, the absorbed dose to the bone marrow originating from a specified source region (*D*_BM ← source_) was calculated according to Eq. (2) [24]:

2 $$ {D}_{\mathrm{BM}\leftarrow \mathrm{source}}={S}_{\mathrm{BM}\leftarrow \mathrm{source}}\bullet {\overset{\sim }{A}}_{\mathrm{source}}={S}_{\mathrm{BM}\leftarrow \mathrm{source}}\bullet {\int}_{0\ \left(\mathrm{injection}\right)}^{\infty }{A}_{\mathrm{source}}(t) dt. $$

${\overset{\sim }{A}}_{\mathrm{source}}$ describes the time-integrated activity per source region and *S*_BM ← source_ the corresponding *S* value or absorbed dose conversion factor. *A*_source_(*t*) corresponds to the source-specific time-activity curve (TAC), which is based on sequential measurements and a subsequent fit to the source time-activity measurements. For this study, *S* values are taken from the public tabulations of Monte Carlo simulation results for the standardised male (Lu-177-PSMA-617 and Lu-177-Octreotate therapy) or female (Lu-177-Octreotate therapy) anthropomorphic phantom as provided, amongst other phantoms, by RADAR [25]. To adjust these phantom-based *S* values to the patient-specific conditions, all *S* values were scaled according to a non-linear mass scaling approach developed by Traino et al. [26].

#### Absorbed dose from the blood time-integrated activity

In the absence of specific binding to the bone marrow or blood cells, as indicated for PSMA therapy [27, 28], the bone marrow self-absorbed dose is solely given by the activity in the extracellular fluid of the marrow tissue [11]. The activity in the extracellular fluid of the bone marrow can be derived from the activity concentration in the blood plasma (blood method), multiplied with the red marrow extracellular fluid fraction (RMECFF = 0.19) of the bone marrow [11, 29, 30]. The activity concentration in the plasma can in turn be determined from the activity concentration in the blood ($\left[{\overset{\sim }{A}}_{\mathrm{blood}}\right]$) and the patient-specific haematocrit (HCT), if there is no specific binding to the blood cells [11]. This yields to:

3.1 $$ {D}_{\mathrm{BM}\leftarrow \mathrm{blood}}^{\mathrm{PSMA}}=\left[{\overset{\sim }{A}}_{\mathrm{blood}}\right]\bullet \mathrm{RMBLR}\bullet {m}_{\mathrm{BM},\mathrm{patient}}\bullet {S}_{\mathrm{BM}\leftarrow \mathrm{BM},\mathrm{phantom}}\bullet {\left(\frac{m_{\mathrm{BM},\mathrm{phantom}}}{m_{\mathrm{BM},\mathrm{patient}}}\right)}^a, $$

3.2 $$ {\mathrm{RMBLR}}^{\mathrm{PSMA}}=\frac{\mathrm{RMECFF}}{1-\mathrm{HCT}}. $$

where RMBLR corresponds to the red-marrow-to-blood activity concentration ratio [11]. *m* denotes the bone marrow (BM) or whole-body (WB) masses (*m*_BM/WB, phantom/patient_) of either the phantom or of the patient [11, 26].

For Lu-177-Octreotate therapy, it holds that:

3.3 $$ {D}_{\mathrm{BM}\leftarrow \mathrm{blood}}^{\mathrm{OCTREO}}=\left[{\overset{\sim }{A}}_{\mathrm{blood}}\right]\bullet \mathrm{RMBLR}\bullet {m}_{\mathrm{BM},\mathrm{patient}}\bullet {S}_{\mathrm{BM}\leftarrow \mathrm{BM},\mathrm{phantom}}\bullet {\left(\frac{m_{\mathrm{BM},\mathrm{phantom}}}{m_{\mathrm{BM},\mathrm{patient}}}\right)}^a. $$

3.4 $$ {\mathrm{RMBLR}}^{\mathrm{OCTREO}}=1 $$

[11, 13]. To scale the male and female *S* values to the patient anatomy, an exponent of *a* = 1.001 and *a* = 0.992 was proposed for Lu-177-PSMA-617 and Lu-177-Octreotate therapy, respectively [26]. To derive the patient-specific blood TAC, 1 ml of blood of each sample was pipetted into a test tube and measured within a Cobra Gamma Counter (Packard Instrument Company, Inc., Meriden, CT), which has been previously calibrated via five 1-ml test samples of known activity concentration. For the calculation of the time-integrated blood activity concentration, a bi-exponential model was fitted to the time-activity data, followed by integration from zero to infinity according to Eq. (2).

#### Absorbed dose from the remainder of body and major organs

Via subtraction of the time-integrated activity in the extracellular fluid and the time-integrated activity of the main accumulating organs from the whole-body, the respective ROB time-integrated activity (${\overset{\sim }{A}}_{\mathrm{ROB}}$) was determined. The whole-body and organ time-integrated activities, ${\overset{\sim }{A}}_{\mathrm{WB}}$ and ${\overset{\sim }{A}}_{\mathrm{organ}}$, were determined from a mono-exponential fit to the three measurement points at 24, 48, and 72 h post-injection. All organ activities were derived from the sequential SPECT images, while for the determination of the whole-body activity, the sequential whole-body planar images were used. The kidneys were considered as main accumulating organs for both Lu-177-Octreotate and Lu-177-PSMA-617 therapy, according to the previous studies assessing dosimetric estimates [5, 7–9]. The patient-specific volumes of interest (VOIs) for the kidneys were defined based on a percent isocontour of the organ maximum and of the quantitative SPECT at 24 h p. i. (PMOD Version 3.609), since images taken at early time points offer a high signal-to-background ratio for organ delineation. We adjusted the isocontour level for each patient in the best way with the usage of the CT as guidance. For all patients, an isocontour level of 30–40% was found to be appropriate. All kidney VOIs were copied to the following SPECT scans 48 and 72 h p. i., which were co-registered onto the SPECT scan 24 h p. i. in advance. We manually re-positioned, i.e. shifted or rotated, the kidney VOIs in case of imperfect co-registration of the individual SPECT time points. For Lu-177-Octreotate therapy, the liver and spleen were additionally included in the bone marrow absorbed dose from the organs [5]. For the patient-wise delineation of the liver and spleen, a similar approach as for the kidney definition was chosen using a 10 to 15% isocontour for the liver and a 30 to 40% isocontour for the spleen. The lower isocontour for liver delineation can be explained by the fact that NET patients often exhibit liver metastases, which lead to a heterogeneous activity accumulation with multiple hot spots.

The bone marrow absorbed dose from the ROB is finally given by the following formula according to Hindorf et al. with adjusted exponents as proposed by Traino et al. [11, 26]:

4.1 $$ {\overset{\sim }{A}}_{\mathrm{ROB}}={\overset{\sim }{A}}_{\mathrm{WB}}-\left[{\overset{\sim }{A}}_{\mathrm{blood}}\right]\bullet \mathrm{RMBLR}\bullet {m}_{\mathrm{BM},\mathrm{patient}}-\sum \limits_{\mathrm{all}\ \mathrm{organs}}{\overset{\sim }{A}}_{\mathrm{organ}}, $$

4.2 $$ {D}_{\mathrm{BM}\leftarrow \mathrm{ROB}}={\overset{\sim }{A}}_{\mathrm{ROB}}\bullet \left({S}_{\mathrm{BM}\leftarrow \mathrm{WB},\mathrm{phantom}}\bullet {\left(\frac{m_{\mathrm{WB},\mathrm{phantom}}}{m_{\mathrm{WB},\mathrm{patient}}}\right)}^b\bullet {\left(\frac{m_{\mathrm{BM},\mathrm{phantom}}}{m_{\mathrm{BM},\mathrm{patient}}}\right)}^c-{S}_{\mathrm{BM}\leftarrow \mathrm{BM},\mathrm{phantom}}\bullet {\left(\frac{m_{\mathrm{BM},\mathrm{phantom}}}{m_{\mathrm{ROB},\mathrm{patient}}}\bullet \frac{m_{\mathrm{BM},\mathrm{phantom}}}{m_{\mathrm{BM},\mathrm{patient}}}\right)}^a-\sum \limits_{\mathrm{all}\ \mathrm{organs}}\ {S}_{\mathrm{BM}\leftarrow \mathrm{organ},\mathrm{phantom}}\bullet \frac{m_{\mathrm{organ},\mathrm{phantom}}}{m_{\mathrm{ROB},\mathrm{patient}}}\bullet \frac{m_{\mathrm{BM},\mathrm{phantom}}}{m_{\mathrm{BM},\mathrm{patient}}}\right). $$

Eq. (4.2) considered all phantom- and patient-specific whole-body, ROB, bone marrow, and organ masses *m*_WB/ROB/BM/organ, phantom/patient_ for *S* value scaling. For male and female patients, *b* = 0.896 and *b* = 0.894 as well as *c* = 0.963 and *c* = 0.970 were used, as proposed by Traino et al. [26]. The bone marrow absorbed dose contribution of each individual organ is given by:

5 $$ {D}_{\mathrm{BM}\leftarrow \mathrm{organ}}={\overset{\sim }{A}}_{\mathrm{organ}}\bullet {S}_{\mathrm{BM}\leftarrow \mathrm{organ},\mathrm{phantom}}\bullet \frac{m_{\mathrm{organ},\mathrm{phantom}}}{m_{\mathrm{organ},\mathrm{patient}}}\bullet \frac{m_{\mathrm{BM},\mathrm{phantom}}}{m_{\mathrm{BM},\mathrm{patient}}}. $$

Due to the high tumour load, as it is frequently observed in Lu-177-PSMA-617 therapy and sometimes in Lu-177-Octreotate therapy, we included all tumour activities in the ROB activity and the ROB *S* value was applied. As all patients investigated for Lu-177-Octreotate therapy suffered from liver metastases, the tumour activities had to be removed from the healthy liver activity for each time point. Therefore, tumour VOIs were delineated on the SPECT 24 h p. i. based on a 40% isocontour and transferred to the following SPECT scans, as it was the case for the determination of the organ activities.

### Hybrid imaging for determination of the ROB cross-absorbed dose to the bone marrow

#### Reference dosimetry protocol

**Fig. 2:**
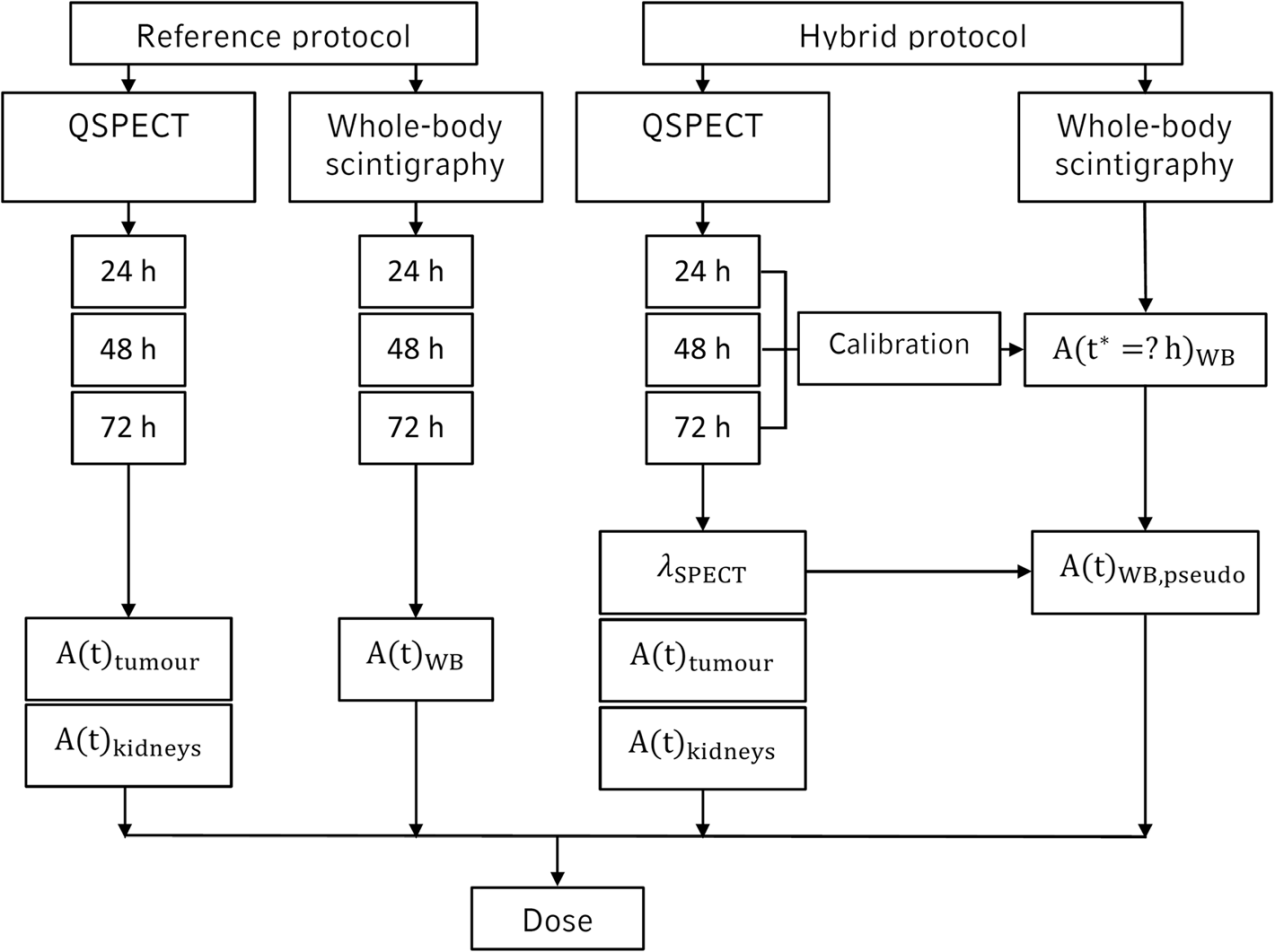
Illustration of the reference protocol (RP) and the proposed hybrid protocol (HP); for the hybrid protocol, the sequential whole-body planar imaging is replaced by a single whole-body planar acquisition at an appropriate time point at 24, 48, or 72 h p. i. (HP24, HP48, HP72); QSPECT indicates quantitative SPECT

For the reference protocol (RP), the bone marrow absorbed dose from the ROB is determined from all three available whole-body planar scans (Fig. 2). For the total bone marrow absorbed dose, the absorbed dose from the three constituents, organs, blood, and ROB, was summed. For each dose constituent, the percentage contribution (PC_constituent_) to the total bone marrow absorbed dose was calculated:

6 $$ {\mathrm{PC}}_{\mathrm{constituent}}=\frac{D_{\mathrm{BM}\leftarrow \mathrm{constituent}}\ }{D_{\mathrm{BM}\leftarrow \mathrm{total}}} \bullet 100\%. $$

#### Hybrid dosimetry protocol

The proposed hybrid protocol (HP) uses a single whole-body image and sequential single-bed quantitative SPECT acquisitions of the abdomen to determine the ROB TAC, instead of deriving the ROB TAC from sequential whole-body planar imaging.

First, the abdominal effective decay constant *λ*_SPECT_ was derived via a mono-exponential fit to the total activity in the SPECT scans 24, 48, and 72 h post-therapy. Especially, all organs and all tumours were included in the fitting of the TAC, as it was the case for the determination of ${\overset{\sim }{A}}_{\mathrm{WB}}$ from the reference protocol. This effective decay constant *λ*_SPECT_ serves as a surrogate for the reference-protocol-based whole-body effective decay constant (Fig. 2). The mono-exponential SPECT-based abdominal TAC was then scaled with a chosen base point. This base point is defined via the whole-body activity *A*_WB_(*t*^∗^) of a single whole-body planar image acquired at an arbitrary time point *t*^∗^∈ 24, 48, or 72 h post-therapy. The resulting pseudo-whole-body TAC *A*_WB, pseudo_(*t*) is intended to serve as an estimate of the reference-protocol-based whole-body TAC (Eq. (7.1)) and can be further used to determine a pseudo-whole-body time-integrated activity${\overset{\sim }{A}}_{\mathrm{WB},\mathrm{pseudo}}$ (Eq. (7.2)).

7.1 $$ {A}_{\mathrm{WB},\mathrm{pseudo}}(t)={A}_{\mathrm{WB},\mathrm{pseudo}}\left({t}^{\ast}\right)\bullet \exp \left(-{\lambda}_{\mathrm{SPECT}}\bullet \left(t-{t}^{\ast}\right)\right), $$

7.2 $$ {\overset{\sim }{A}}_{\mathrm{WB},\mathrm{pseudo}}={\int}_{t=0}^{\infty }{A}_{\mathrm{WB},\mathrm{pseudo}}\left({t}^{\prime}\right)d{t}^{\prime }=\frac{A_{\mathrm{WB}}\left({t}^{\ast}\right)\bullet \exp \left({\lambda}_{\mathrm{SPECT}}\bullet {t}^{\ast}\right)}{\lambda_{\mathrm{SPECT}}}=\frac{A_{\mathrm{WB}}\left({t}^{\ast}\right)\bullet \exp \left({\lambda}_{\mathrm{SPECT}}\bullet {t}^{\ast}\right)\bullet {T}_{1/2,\mathrm{SPECT}}}{\ln (2)}. $$

*T*_1/2, SPECT_ denotes the SPECT-based effective half-life.

#### Comparison of reference and hybrid absorbed dose values

Based on the hybrid model given in Eqs. (7.1) and (7.2), the bone marrow absorbed dose from the ROB can be estimated by Eqs. (4.1) and (4.2). In this work, we investigated a combination of the sequential abdominal SPECT with the whole-body planar images at 24, 48, or 72 h p. i., where each whole-body planar image was individually calibrated via the quantitative SPECT at the corresponding time point (Fig. 2). These different hybrid protocols were further denoted as HP24, HP48, and HP72. The agreement of the bone marrow absorbed doses from the ROB, as determined via the HP and the RP, was assessed. Therefore, the percentage deviation between absorbed dose estimates (PD_dose_; Eq. (8)) was calculated, and a statistical test for correlation was performed (MATLAB Pearson correlation analysis).

8 $$ {\mathrm{PD}}_{\mathrm{dose}}=\left|\left(\frac{D_{\mathrm{HP}24/\mathrm{HP}48/\mathrm{HP}72}-{D}_{\mathrm{RP}}}{D_{\mathrm{RP}}}\right)\right|\bullet 100\%. $$

Furthermore, the same analysis was performed regarding the total bone marrow absorbed dose estimates composed of all available constituents: the ROB (including tumours), the explicitly analysed organs, and the contribution of the blood activity. While the application of the hybrid protocol affects the bone marrow absorbed dose from the ROB, all other constituents were not altered.

#### Comparison of hybrid and reference ROB TAC parameters

For a mono-exponential TAC, the time-integrated activity is calculated as the product of the effective half-life *T*_1/2_ and the *y*-axis intercept *A*_0_ of the fit function:

9 $$ \overset{\sim }{A}=\frac{A_0}{\ln (2)}\bullet {T}_{1/2.} $$

The proposed hybrid protocol assumes that ideally, the SPECT-based abdominal effective half-life is equal to the whole-body effective half-life. However, in reality, differences in both half-lives will lead to deviations in the area under the whole-body TACs derived from the reference protocol and hybrid protocol, and thus in the respective whole-body and ROB time-integrated activities. Simultaneously, these deviations in the course of the TACs may affect the *y*-axis intercepts of the reference-protocol-based and hybrid-protocol-based TACs. To address this issue, both fit function parameters, effective half-life and the *y*-axis intercept, were compared for the reference protocol, HP24, HP48, and HP72. For a perfect agreement between the reference-protocol-based and hybrid-protocol-based ROB time-integrated activities, ${\overset{\sim }{A}}_{\mathrm{RP}}$ and ${\overset{\sim }{A}}_{\mathrm{HP}}$, the product of the ratio of reference-to-hybrid effective half-lives $\left(\frac{T_{\mathbf{1}/\mathbf{2},\mathrm{RP}}}{T_{\mathbf{1}/\mathbf{2},\mathrm{HP}}}\right)$ and the ratio of reference-to-hybrid *y*-axis intercepts $\left(\frac{A_{0,\mathrm{RP}}}{A_{0,\mathrm{HP}}}\right)$ has to yield 1:

10 $$ \frac{{\overset{\sim }{A}}_{\mathrm{RP}}}{{\overset{\sim }{A}}_{\mathrm{HP}}}=\frac{A_{0,\mathrm{RP}}}{A_{0,\mathrm{HP}}}\bullet \frac{T_{\mathbf{1}/\mathbf{2},\mathrm{RP}}}{T_{\mathbf{1}/\mathbf{2},\mathrm{HP}}}=1 $$

## Results

### Reference dosimetry protocol

**Fig. 3:**
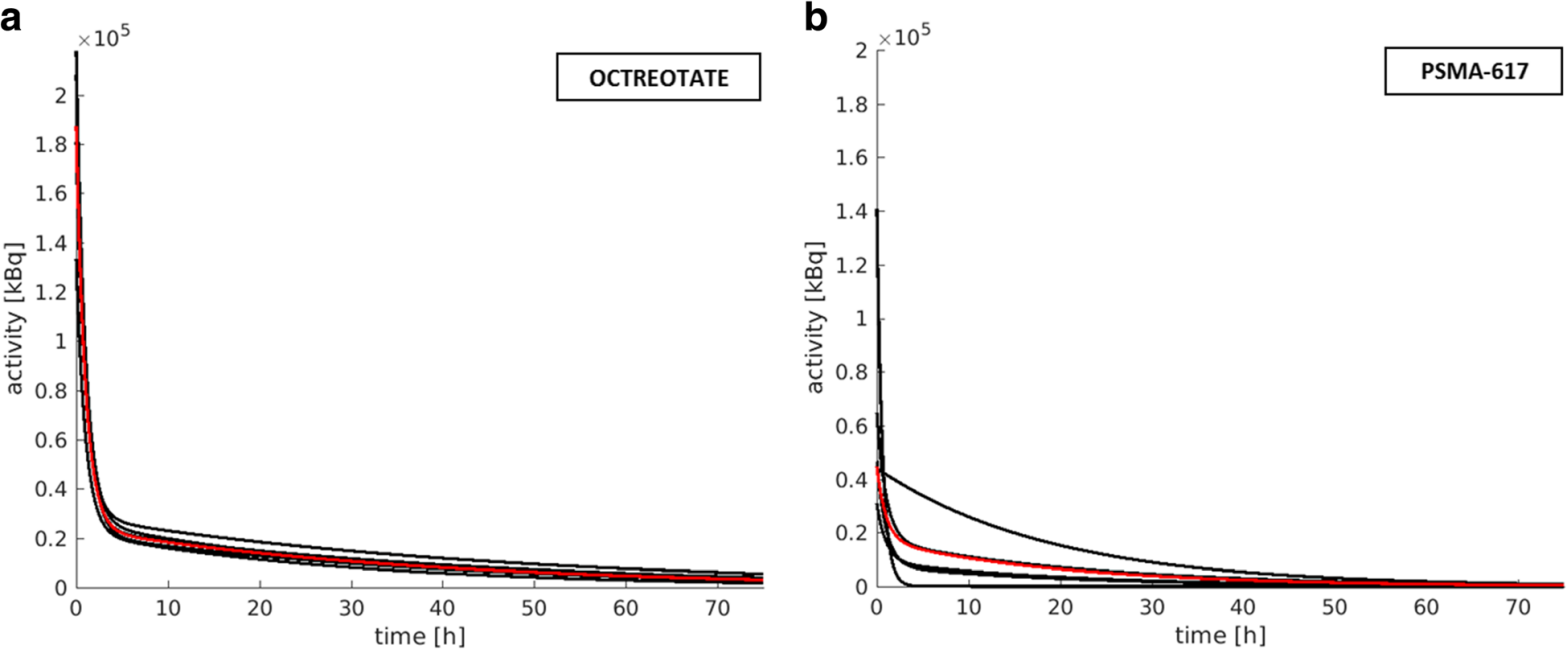
Time-activity curves (TAC) for the bone marrow self-absorbed dose as determined via the blood method and for both Lu-177-Octreotate (**a**) and Lu-177-PSMA-617 therapy (**b**) (according to Eqs. (3.1) and (3.3)); the patient-specific TACs are shown in black, while the median is presented in red

Based on the reference protocol, median total bone marrow absorbed doses were calculated as 12.1 mGy/GBq (range 9.6–15.6 mGy/GBq) for Lu-177-Octreotate and 10.8 mGy/GBq (range 6.7–16.8 mGy/GBq) for Lu-177-PSMA-617 therapy (Table 3). The blood absorbed dose contribution was higher for Lu-177-Octreotate compared with Lu-177-PSMA-617 therapy, with a larger inter-patient variability for Lu-177-PSMA-617 therapy. The median values were found to be 59% (range 50–63%) for Lu-177-Octreotate therapy and 43% (range 13–63%) for Lu-177-PSMA-617, respectively (Table 3). The median ROB contribution was 34% (range 29–41%) for Lu-177-Octreotate and 45% (range 34–80%) for Lu-177-PSMA-617 therapy, again with a higher variance of the patient-specific percentage contributions for Lu-177-PSMA-617 therapy (Table 3). For Lu-177-PSMA-617 therapy, the higher percentage ROB contribution to the total bone marrow absorbed dose is on the one hand driven by the larger tumour load for the investigated mCRPC patients, as all tumours were included in the ROB compartment. On the other hand, the percentage contribution of the bone marrow absorbed dose from the blood is reduced for Lu-177-PSMA-617 therapy compared with Lu-177-Octreotate therapy due to the weighting of the blood activity with the patient haematocrit and the RMECFF, yielding an average weighting factor of 0.3. Furthermore, for the five mCRPC patients in this study, a lower median effective half-life for the slow phase of the bi-exponential fit to the blood time-activity measurements was observed, compared with the five NET patients (Lu-177-Octreotate, 25 h; Lu-177-PSMA-617, 14 h; Fig. 3). The major accumulating organs contributed at maximum 9% (median: all organs 8%, kidneys 4%, liver 2%, spleen 1%) for Lu-177-Octreotate therapy and 12% (median kidneys 8%) for Lu-177-PSMA-617 therapy (Table 3).

**Table 3:** Results from the reference protocol. Total bone marrow absorbed doses (*D*_BM ← total_) and the percentage contribution of ROB, blood, and organs to the total bone marrow absorbed dose. All percentage contributions were calculated according to Eq. (6)

Patient	*D*_BM ← total_ [mGy/GBq]	ROB [%]	Blood [%]	Organs [%]
Octreotate				
P1	12.1	29	63	8
P2	9.6	34	60	6
P3	15.6	34	59	7
P4	11.8	36	59	5
P5	12.7	41	50	9
Median	12.1	34	59	8
PSMA-617				
P6	10.2	36	56	8
P7	6.7	45	43	12
P8	16.8	80	13	7
P9	14.2	34	63	3
P10	8.3	60	28	12
Median	10.8	45	43	8

### Hybrid protocol and comparison of reference and hybrid absorbed dose values

**Fig. 4:**
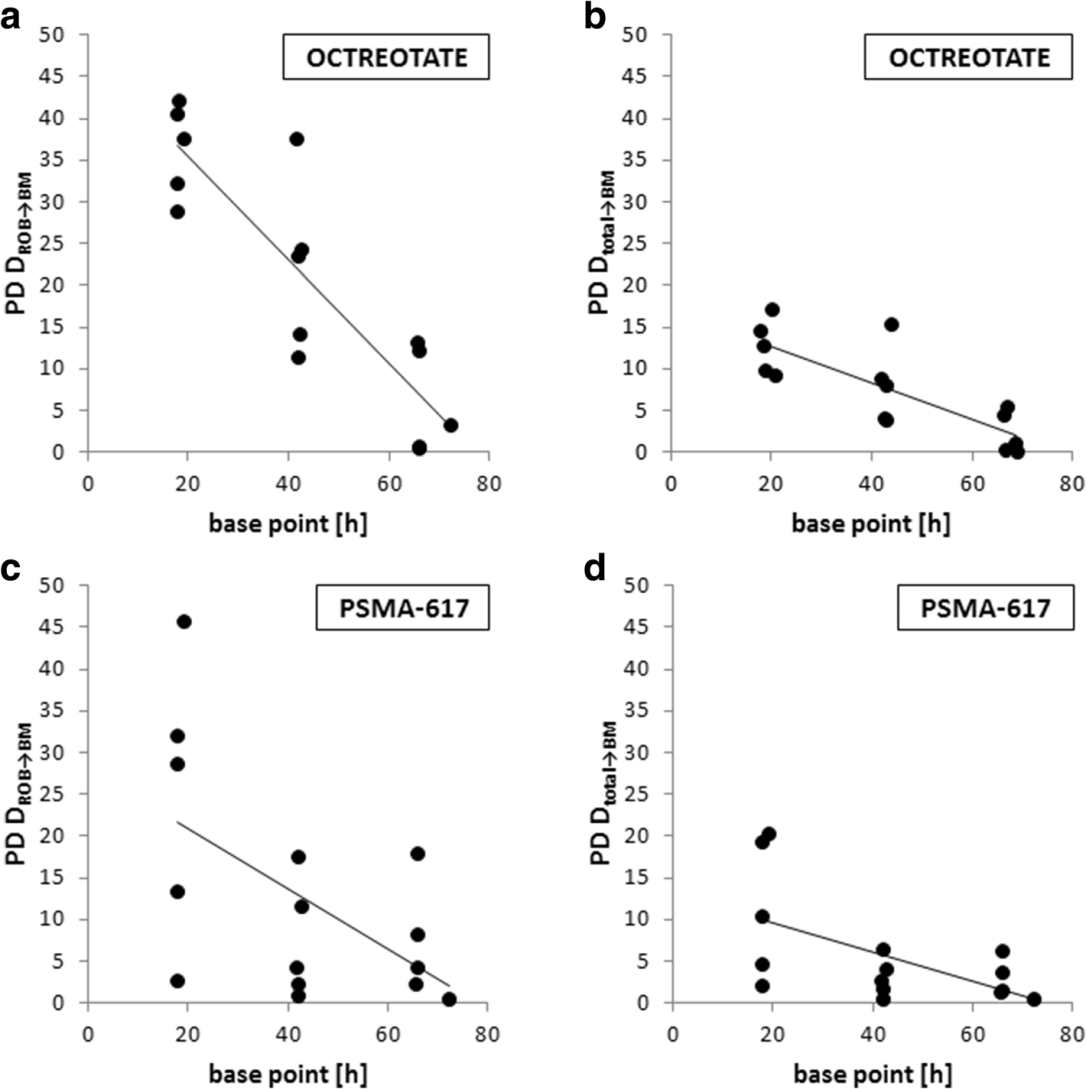
Percentage deviation (PD) from the reference protocol for the bone marrow absorbed dose from the ROB and the total bone marrow absorbed dose depending on the base point used for the hybrid protocol (24, 48, or 72 h p. i.). All percentage deviations were calculated according to Eq. (8)

**Fig. 5:**
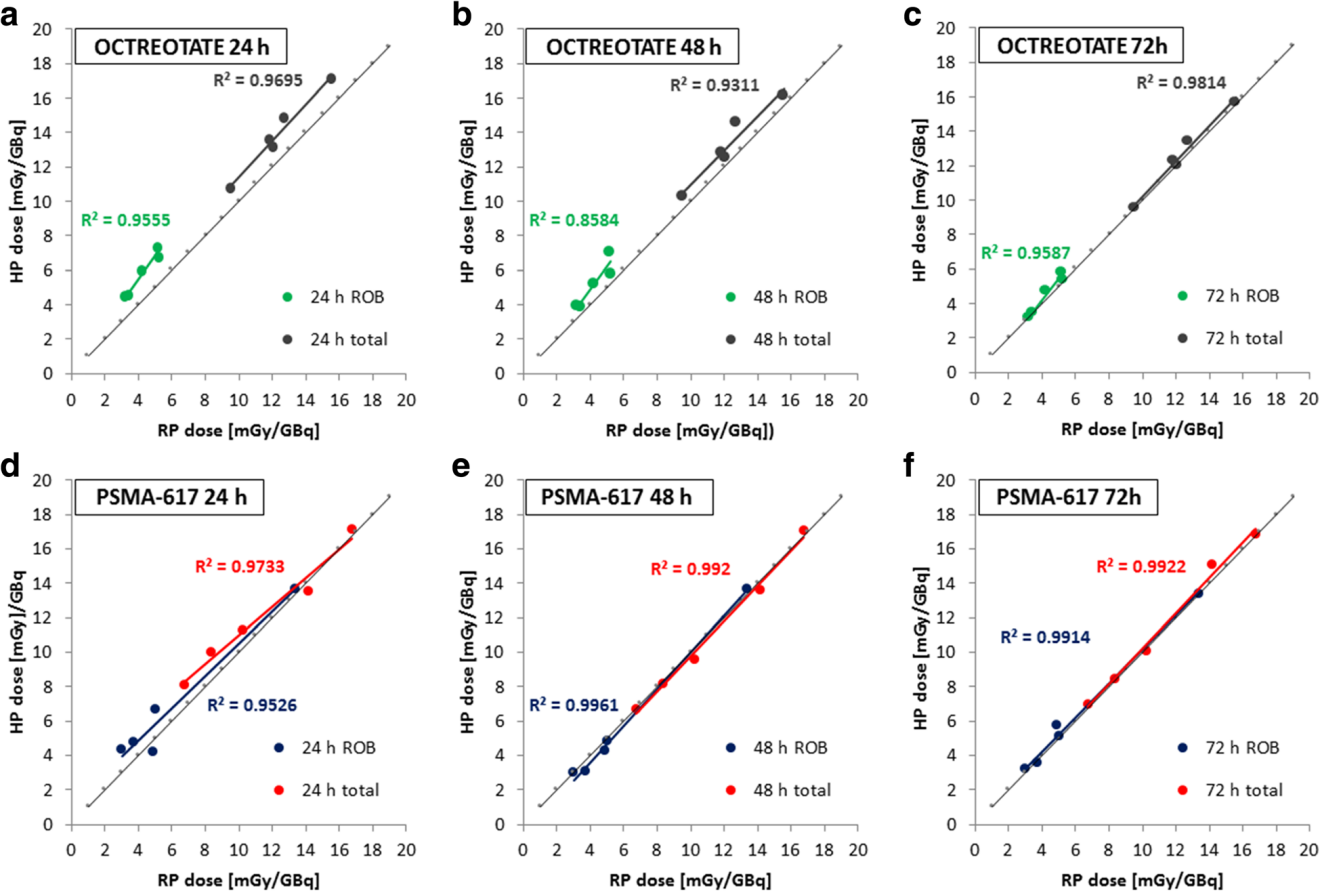
Comparison of bone marrow absorbed doses from the ROB and total bone marrow absorbed doses as calculated via the reference protocol and the HP24, HP48, and HP72 (24, 48, or 72 h p. i.)

For Lu-177-Octreotate therapy, the median deviations of the bone marrow absorbed dose from the ROB were found to be 37% (range 29–42%), 23% (range 11–38%), and 3% (range 1–13%) for the HP24, HP48, and HP72, respectively, compared with the results obtained via the reference protocol (Fig. 4a). A very strong and significant (*p* < 0.05) correlation between the reference and hybrid protocol was confirmed for all base points 24, 48, 72 h p. i. with Pearson correlation coefficients of 0.98, 0.93, and 0.98. However, a tendency of overestimation of the bone marrow absorbed dose from the ROB, especially for the HP24 and the HP48, is noticed (Fig. 5a–c). The respective deviations for Lu-177-PSMA-617 were found to be 29% (range 3–46%), 4% (range 1–17%), and 4% (range 1–18%) (Fig. 4c) with a very strong Pearson correlation of 0.98, 1.00, and 1.00, respectively (Fig. 5d–f). The tendency of overestimation of the bone marrow absorbed dose from the ROB was also evident for the HP24, but reduced for the HP48 and HP72 (Fig. 5d–f).

The deviations between the reference and hybrid protocol were lower for the total bone marrow absorbed dose estimates compared with those for the ROB alone (Fig. 4). For Lu-177-Octreotate therapy, median differences of the total bone marrow absorbed doses were 13% (range 9–17%), 8% (range 4–15%), and 1% (range 0–5%) using the HP24, HP48, and HP72, respectively, with a very strong and significant (*p* < 0.05) Pearson correlation of 0.98, 0.96, and 0.99 (Figs. 4b and 5a–c). As it was the case for the bone marrow absorbed dose from the ROB alone, especially the use of an early base point leads to overestimated absorbed dose values (Table 4 and Fig. 5a–c). For Lu-177-PSMA-617 therapy, the median deviations were found to be 10% (range 2–20%), 3% (range 0–6%), and 2% (range 0–6%) with a very strong correlation of 0.99, 1.00, and 1.00, respectively (Figs. 4d and 5d–f). The tendency of overestimated absorbed dose values was particularly evident for the base point 24 h p. i. (Table 4 and Fig. 5d–f).

**Table 4:** Comparison of the reference and hybrid protocol for different time points of single whole-body planar image acquisition (24 h p. i.: HP24; 48 h p. i.: HP48; 72 h p. i.: HP72); all calculated total bone marrow absorbed doses (*D*_BM ← total_) are provided

Patient	*D*_BM ← total_ RP [mGy/GBq]	*D*_BM ← total_ HP24 [mGy/GBq]	*D*_BM ← total_ HP48 [mGy/GBq]	*D*_BM ← total_ HP72 [mGy/GBq]
Octreotate				
P1	12.1	13.2	12.6	12.1
P2	9.6	10.8	10.3	9.6
P3	15.6	17.1	16.2	15.7
P4	11.8	13.5	12.8	12.3
P5	12.7	14.9	14.7	13.4
Median	12.1	13.5	12.8	12.3
PSMA-617				
P6	10.2	11.3	9.6	10.1
P7	6.7	8.1	6.7	7.0
P8	16.8	17.1	17.1	16.8
P9	14.2	13.5	13.6	15.1
P10	8.3	10.0	8.1	8.5
Median	10.2	11.3	9.6	10.1

To summarise, for Lu-177-Octreotate, the best agreement with respect to the reference protocol was obtained with the hybrid protocol based on 72 h p. i. for all patient cases, while for Lu-177-PSMA-617 therapy for 40% of the patients, the time point of 48 h p. i. and for 40% the acquisition of 72 h p. i. was best suited. For one mCRPC patient, both base points, 48 and 72 h p. i., provided the same absolute deviation from the reference (Table 4).

### Comparison of hybrid and reference TAC parameters

For both Lu-177-Octreotate and Lu-177-PSMA-617 therapy, the whole-body effective half-life was shorter compared with the washout in the abdominal region, except for patient P9, who presented with pronounced and strongly accumulating bone metastasis in the right hip (Table 5). Median whole-body and abdominal effective half-lives were found to be 43 h (range 40–62 h) and 61 h (range 53–87 h) for Lu-177-Octreotate therapy and 31 h (range 22–65 h) and 42 h (range 31–67 h) for Lu-177-PSMA-617 therapy. Table 5 indicates a tendency to lower whole-body and abdominal effective half-lives for Lu-177-PSMA-617 compared with Lu-177-Octreotate therapy, except for patient P8, who showed the highest bone tumour load with strong and persistent retention of the radiopharmaceutical (Fig. 1). The deviation between the effective half-lives was similar for both therapies with 40% (range 30–42%) for Lu-177-Octreotate therapy and 46% (range 4–64%) for Lu-177-PSMA-617 therapy, however, with a larger observed variability for Lu-177-PSMA-617 therapy (Table 5).

**Table 5:** Comparison of planar-based whole-body (*T*_1/2, WB_) and SPECT-based abdominal effective half-lives (*T*_1/2, SPECT_) for Lu-177-PSMA-617 and Lu-177-Octreotate therapy

Patient	*T*_1/2, WB_ [h]	*T*_1/2, SPECT_ [h]
Octreotate		
P1	40	53
P2	43	61
P3	43	56
P4	52	73
P5	62	87
Median	43	61
PSMA-617		
P6	22	33
P7	31	50
P8	65	67
P9	39	31
P10	29	42
Median	31	42

**Fig. 6:**
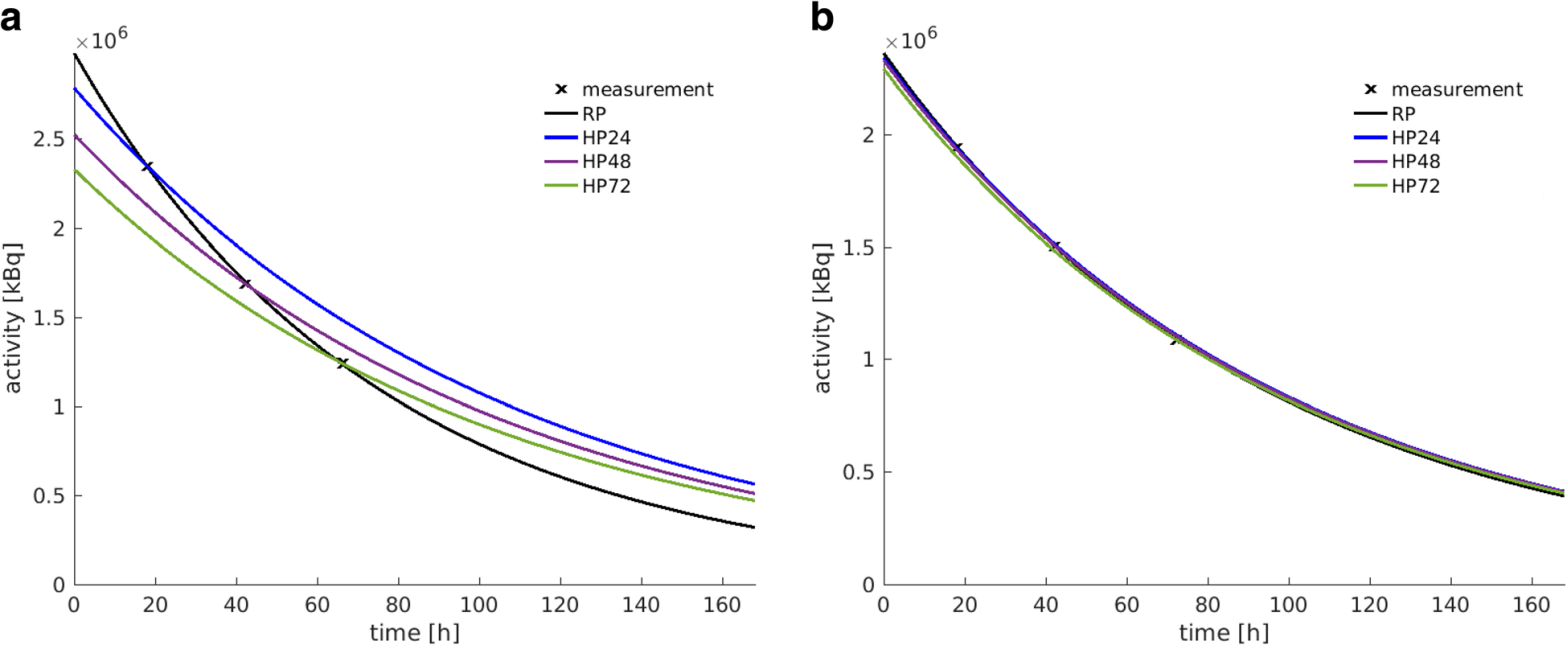
Whole-body TACs for (**a**) patient P4 (Lu-177-Octreotate) and (**b**) patient P8 (Lu-177-PSMA-617) to visualise the impact of the time point of whole-body planar image acquisition during hybrid-protocol-based calculation of the bone marrow absorbed dose from the ROB (24, 48, or 72 h p. i.)

**Fig. 7:**
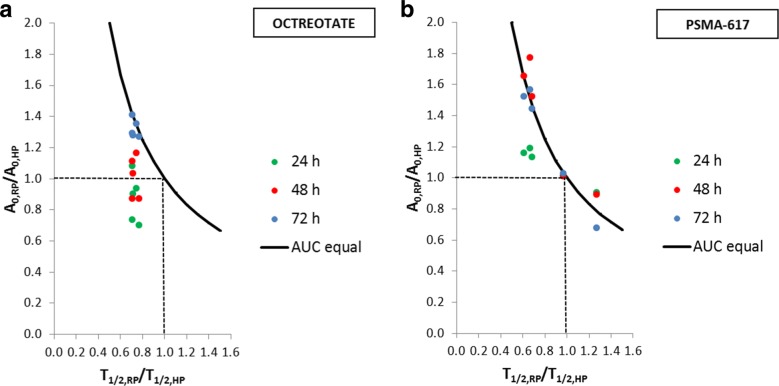
Evaluation of hybrid-protocol-based TAC parameters in comparison to the reference protocol for both therapies; the ratio of the reference-protocol-based and SPECT-based effective half-lives (*T*_1/2,RP_/*T*_1/2,HP_) and the ratio of the *y*-axis intercepts for the reference-protocol-based and the hybrid-protocol-based TACs (*A*_0,RP_/*A*_0,HP_) are provided; the black curve indicates the optimal case, for which the area under both TACs (AUC) is equal

Figure 6 shows examples of fitted reference-protocol-based whole-body and hybrid-protocol-based whole-body TACs for both Lu-177-Octreotate (patient P4) and Lu-177-PSMA-617 (patient P8) therapy. The use of the SPECT-based effective half-life for the hybrid protocol leads to an under- and overestimation of the reference-protocol-based TAC before and after the selected base point. This under- and overestimation is varying for the HP24, HP48, and HP72 and also affects the *y*-axis intercept of the hybrid-protocol-based TACs in comparison to the reference protocol. Figure 7 summarises the patient-specific ratios of the reference-to-hybrid effective half-lives in comparison to the ratio of the corresponding *y*-axis intercepts. The black line indicates all combinations of effective half-life and *y*-axis intercept ratios, for which the reference-protocol-based and hybrid-protocol-based time-integrated activities are equal. For Lu-177-Octreotate therapy, the median ratio of the reference-to-hybrid effective half-lives was found to be 0.7 (range 0.7–0.8). Simultaneously, the reference-to-hybrid *y*-axis intercept ratios increase for the base points from 24 to 72 h post-therapy. For the HP72, the combination of effective half-life and *y*-axis intercept ratios yields to the closest agreement between the reference-protocol-based and hybrid-protocol-based time-integrated activities (Fig. 7a). For Lu-177-PSMA-617 therapy, the median ratio of the reference-to-hybrid effective half-lives was calculated as 0.7 (range 0.6–1.3). The larger variability in the reference-to-hybrid effective half-life ratios is also evident in Fig. 7b. For Lu-177-PSMA-617 therapy, for the time points 48 and 72 h p. i., combinations of reference-to-hybrid effective half-life ratios and *y*-axis intercept ratios were found which result close to a ratio of 1between the reference-protocol-based and hybrid-protocol-based ROB time-integrated activities.

## Discussion

Although all bone marrow absorbed dose estimates are well below the typically applied critical threshold of 2 Gy [5] and no severe marrow toxicities have been observed for all investigated patients, bone marrow dosimetry is still a matter of interest. This is particularly true regarding the maximum absorbed dose that can be applied for patients with progressive cancer disease, who already underwent several pre-therapies. The absorbed dose estimates determined in this study are in good agreement with the findings of previous studies for both therapies [5, 7, 8, 31].

According to the current clinical standard, an uncertainty of at least 10–20% has to be expected for the derived activity and absorbed dose values in case of quantitative Lu-177 SPECT imaging, and even greater values might be expected for planar imaging [15–17, 32, 33]. Thus, the results presented in this study suggest that the application of a hybrid SPECT planar dosimetry approach based on late whole-body planar images allows for bone marrow dosimetry which is sufficiently reliable and applicable in clinical routine. In the case of Lu-177-Octreotate therapy of patients bearing NET and with regard to our institutional measurement protocol, the best time point for whole-body planar imaging was found to be approximately at 72 h p. i., with maximum deviations of the total bone marrow absorbed dose of 5% compared to the reference protocol. In patients with mCRPC receiving Lu-177-PSMA-617 therapy, the whole-body planar imaging time points 48 and 72 h p. i. provided comparable total bone marrow absorbed dose estimates with similar maximum differences of 6% to the reference-protocol-based full sequential whole-body planar approach. If five to ten Lu-177-PSMA-617 or Lu-177-Octreotate therapies are offered per week, the reduction of whole-body planar scans from three to one results in a reduction of examination time of 3.5 to 7 h per week. Simultaneously, the application of the proposed hybrid imaging protocol does not lead to an increased workload for the absorbed dose calculations.

The magnitude of deviations depends on the differences in the abdominal and whole-body washout and the positioning of the base point used for scaling of the mono-exponential pseudo-whole-body TAC. Analysis of the patient-specific reference-protocol-based and hybrid-protocol-based TAC parameters revealed that the use of a prolonged SPECT-based effective half-life is compensated by a lower *y*-axis intercept, if a later base point is selected. The use of a base point later than 72 h p. i. still has to be investigated; however, such a time point was unfortunately not available in our institutional measurement protocol. As expected, the deviations between the reference and hybrid protocol were larger for the bone marrow absorbed dose from the ROB compared with the total bone marrow absorbed dose, as the median ROB contribution to the total absorbed dose was found to be only 34% for Lu-177-Octreotate therapy and 45% for Lu-177-PSMA-617 therapy.

The appropriate whole-body planar imaging time point may have to be determined separately for each type of therapy. The degree of the deviations between abdominal and whole-body effective decay constants is driven by the disease- or therapy-specific retention in the organs and tumours and the corresponding typical tumour distribution. The mCRPC patients included in this study typically showed a larger tumour load compared with the NET patients, which was additionally strongly varying over the whole patient body. For most of the mCRPC patients (except P9) included in this study, the main metastatatic load was located in the torso, and consequently, the abdominal effective half-life was larger compared with the whole-body effective half-life. By contrast, patient P9 suffered from a strongly accumulating metastasis in the hip, leading to a comparatively larger whole-body effective half-life. The larger variability in the whole-body tumour distribution for mCRPC patients causes the observed larger spread in the differences between abdominal and whole-body effective half-lives. Consequently, a high tumour load outside the SPECT field of view might lead to an increased uncertainty of the proposed hybrid protocol, and this effect should be further investigated. As it was the case for most of the mCRPC patients, the investigated NET cases mainly presented with metastases in the torso, which lead to an increased retention of the radiopharmaceutical in the abdomen. However, due to the lower tumour load, the inter-patient variability in the abdominal and whole-body effective half-lives was reduced for the NET patients under study.

The change from one-bed abdominal SPECT imaging to the imaging of two or more beds could principally improve the proposed hybrid protocol for bone marrow dosimetry, as an enlarged acquisition area will lead to a more realistic estimate of the whole-body effective half-life. Furthermore, the introduction of fast multi-bed SPECT imaging in the clinical routine would be beneficial for a robust tumour and organ dosimetry over a larger part of the patient body [15–18]. Attempts to introduce fast whole-body SPECT imaging into the clinic already exist [34]. However, the effect of a reduction of scan time on absorbed dose estimates for Lu-177 therapy still has to be evaluated.

The accuracy of dosimetry based on standardised organ-level *S* values is limited, as such *S* values are inherently not capable to fully consider the patient-specific full 3D functional and anatomical characteristics. The latter fact remains true, even if a scaling of the *S* values to the specific anatomical conditions is applied [6, 14, 35–37]. For Lu-177, the ROB cross-absorbed dose of the bone marrow is mainly driven by the long-range photon component, which is more sensitive to the anatomy than the locally deposited beta absorbed dose. In a previous study based on Monte Carlo simulations, deviations of the order of up to 100% were observed, if photon cross-absorbed doses were calculated based on standardised *S* values [38]. Furthermore, *S* values are determined based on the assumption of homogeneous activity accumulation. However, the activity accumulation in the ROB with the inclusion of tumours is highly heterogeneous with the degree of heterogeneity being caused by both tumour load and distribution. With regard to both aspects the limited consideration of the patient-specific functional and anatomical characteristics, the reliablity of the proposed hybrid protocol can be well accepted in the framework of organ-level *S* values. Moreover, it should also be noted that the exact bone marrow distribution of each patient is a priori unknown due to the heterogeneous micro-structure of the bone marrow and its pathologically highly variable distribution, which both lead to a highly unspecified target for bone marrow dosimetry [23]. Particularly, for mCRPC patients with a high bone tumour load, a displacement of active bone marrow from highly metastasised to tumour-free skeletal sites is possible [39].

Our decision to include all tumours in the ROB represents a simplified approach for clinical routine bone marrow dosimetry. On the one hand, this approach is more practical, as in case of a high bone tumour load, a manual determination of the time-integrated activity is not feasible for each tumour lesion in an acceptable time. On the other hand, even if a semi-automatic or automatic tumour segmentation is available, tumour-to-bone marrow *S* values for both individual tumours and the total tumour distribution are not available, as tumours are quite variable in shape, size, and position, and the pre-calculation of all possible *S* values is not possible. Thus, at this point, a more simplified approach was chosen, which considered all tumours at once within the ROB compartment. The approximation to use the *S* value of the compartment in which the tumours are located to estimate the bone marrow absorbed dose from lesions has also been applied in previous studies [5]. An alternative way, proposed by Svensson et al. for bone marrow dosimetry for Lu-177-Octreotate therapy, differentiates the activity distribution in the patient body in low- and high-activity regions (background vs. main accumulating organs and tumours) with separate *S* values applied to each of both compartments [31]. The resulting bone marrow absorbed doses correlated with the change of blood parameters and were found to be in a similar range compared to previously published results. Monte Carlo studies may help in further understanding the effect of such simplifying assumptions for bone marrow dosimetry.

## Conclusions

For both Lu-177-PSMA-617 and Lu-177-Octreotate therapy, bone marrow dosimetry can be performed via a single whole-body planar image and a sequential SPECT (hybrid protocol), provided that this planar image is acquired at a later time point. Regarding the three imaging time points 24, 48, and 72 h, which were available for this study, the time points of 48 or 72 h p. i. were found to be suitable for Lu-177-PSMA-617 therapy. For Lu-177-Octreotate therapy, a time point of 72 h p. i. was identified as appropriate. This hybrid protocol enables total bone marrow absorbed dose estimates with a maximum deviation of 5–6% compared to a dosimetry protocol using both sequential planar and SPECT imaging. These deviations can be considered acceptable with regard to the uncertainties which currently have to be expected for Lu-177 quantitative imaging and bone marrow dosimetry based on organ-level *S* values. However, the proposed hybrid protocol allows for a more patient-friendly and time-efficient bone marrow dosimetry in clinical routine due to the decreased examination times.
